# US costs and outcomes associated with Clostridium difficile infections: a systematic literature review, meta-analysis, and mathematical model

**DOI:** 10.1186/2047-2994-4-S1-O37

**Published:** 2015-06-16

**Authors:** ML Schweizer, R Nelson, M Samore, S Nelson, K Khader, H-Y Chiang, M Chorazy, L Herwaldt, D Diekema, A Blevins, M Ward, E Perencevich

**Affiliations:** 1 Internal Medicine, University of Iowa, Iowa City, IA, USA; 2 Internal Medicine, University of Utah, Salt Lake City, UT, USA; 3 Epidemiology, University of Iowa, Iowa City, IA, USA; 4 Library Sciences, University of Iowa, Iowa City, IA, USA

## Introduction

An understanding of the health and economic impact of *C. difficile* infections (CDI) can inform investments in prevention and treatment interventions.

## Objectives

To estimate the burden of CDI in the US using a meta-analysis and economic model.

## Methods

We searched PubMed, CINAHL, EMBASE and others for multicenter studies published in the US between 2000-2014 that evaluated CDI outcomes or costs. Studies were included in the economic analysis if they measured post-infection costs, post-infection length of stay (LOS), or propensity score-matched CDI patients to non-CDI controls. We also included studies that evaluated CDI-associated mortality with a control group. We created an economic model using TreeAgePro 2014.

## Results

When the 22 studies that evaluated mortality were pooled, CDI was associated with a 2.5-fold increase in mortality compared with other hospitalized patients (pooled RR=2.54; 95% CI: 1.89, 3.40). Only 4 low/moderate quality studies evaluated costs of CDI. The mean CDI-attributable cost of the index hospitalization ranged from $8,426 to $48,500. The mean costs per CDI after discharge were $1,592 for outpatient visits and $14,847 for readmissions. When these values were adjusted to 2013 US dollars and included in the economic model, we found that the mean total cost of a CDI was $32,198 (SD =$9,798). Of the 3 studies that evaluated LOS using propensity matching, the mean CDI-attributable LOS was 12.3 days. When this excess LOS was multiplied by an average cost per day from a private 3^rd^ party payer perspective, CDI cost an average of $56,663 (SD =$19,804).

## Conclusion

Pooled estimates from the currently available literature suggest that CDI is associated with large health and economic burdens. However, the majority of available studies were of moderate/low quality and may overestimate the outcomes. Thus, these estimates should be used with caution and higher-quality studies should be completed to guide future economic evaluations of CDI prevention and treatment interventions.

## Disclosure of interest

None declared.

